# Machine Learning–Based Prediction Model for 30-Day Emergency Department Revisits in a Medically Underserved Tertiary Hospital: Formative Retrospective Cohort Study

**DOI:** 10.2196/87289

**Published:** 2026-05-29

**Authors:** Kyongmin Sun

**Affiliations:** 1 Department of Emergency Medicine, College of Medicine, Gangneung Asan Hospital, Gangneung, Gangwon-do, Republic of Korea; 2 Department of Health Administration, Graduate School, Yonsei University Mirae Campus, 1 Yonsedae-gil, Heungeop-myeon, Wonju, Gangwon-do, 26493, Republic of Korea, 82 337602418

**Keywords:** emergency department, revisit, machine learning, extreme gradient boosting, XGBoost, Shapley additive explanations, SHAP, prediction model, medically underserved area

## Abstract

**Background:**

Emergency department (ED) revisits are critical quality indicators, particularly in medically underserved areas, where traditional prediction tools show limited performance. Machine learning (ML) approaches may offer improved predictive performance for identifying high-risk patients.

**Objective:**

This formative study aimed to develop and validate an ML-based model for predicting 30-day ED revisits using electronic health records from a tertiary hospital serving a medically underserved area in South Korea and to evaluate its clinical utility through interpretability analysis and risk stratification.

**Methods:**

This retrospective cohort study analyzed 36,230 adult patients visiting the Gangneung Asan Hospital ED in 2023. We developed and compared 3 ML models (extreme gradient boosting [XGBoost], random forest, and ElasticNet) using electronic health records. Model interpretability was ensured through Shapley additive explanations (SHAP) analysis, and clinical utility was evaluated through 5-tier risk stratification.

**Results:**

Among 36,230 patients, 798 (2.2%) revisited within 30 days. XGBoost achieved superior performance with an area under the receiver operating characteristic curve of 0.90 (95% CI 0.88‐0.92), a sensitivity of 0.94, and a specificity of 0.69. The SHAP analysis identified ED length of stay, oxygen saturation, systolic blood pressure, computed tomography performance, antibiotic use, and liver disease as key predictors. Risk stratification demonstrated a 25-fold difference in the actual revisit rates between the lowest (152/8450, 1.8%) and the highest (686/1500, 45.7%) risk groups.

**Conclusions:**

The XGBoost model demonstrated excellent predictive performance with high interpretability for 30-day ED revisit predictions. The implementation of this model could enable risk-stratified interventions and more efficient resource allocation in medically underserved settings, potentially reducing unnecessary revisits and improving patient outcomes. This formative study establishes feasibility and provides a foundation for future multicenter validation studies in similar medically underserved settings.

## Introduction

Emergency department (ED) revisits are globally recognized as key indicators of health care quality. Revisits within 30 days may reflect inadequate initial treatment, failure of postdischarge management, or deterioration of underlying conditions, with significant implications for patient safety and health care costs. This issue is particularly severe in medically underserved regions, where tertiary hospitals serving extensive catchment areas often face challenges in appropriate postdischarge management owing to patient travel from distant locations and insufficient primary care infrastructure.

Traditional readmission prediction tools, such as the LACE index and HOSPITAL scores, have several fundamental limitations. First, they use only a limited number of variables and fail to adequately reflect patients’ complex clinical conditions. Second, they cannot consider nonlinear relationships or complex interactions between variables, resulting in limited predictive power. Third, their predictive ability is particularly low in ED settings, which reduces their utility in actual clinical practice [1]. Studies have shown that these traditional tools achieve area under the receiver operating characteristic curve (AUROC) values of around 0.59 in older patients, confirming their limitations [1,2].

To overcome these limitations, recent attempts have been made to develop prediction models using vast amounts of electronic medical record (EMR) data and machine learning (ML) techniques. ML offers advantages in learning complex patterns from high-dimensional data and effectively modeling nonlinear relationships between variables [3]. Several successful implementations have demonstrated the potential of this approach. A UK study developed an ED revisit prediction model using extreme gradient boosting (XGBoost) and Shapley additive explanations (SHAP), achieving an AUROC of 0.747 for 72-hour revisit prediction [4]. In the United States, a prediction model incorporating frailty and multimorbidity information using multi-institutional data was developed, with the CatBoost model achieving an AUROC of 0.80 [5]. A large-scale Japanese study analyzed 457,587 admission records from 38 hospitals, systematically comparing ML and traditional logistic regression model performance, with gradient boosting decision tree showing the best performance at an AUROC of 0.764 [6]. A UK Biobank–based study achieved an AUROC of 0.849 with an XGBoost model while ensuring excellent interpretability through the parallel use of SHAP and accumulated local effects [7]. Asian regional studies have shown promising results, with research on older Chinese patients identifying frailty index, polypharmacy, and BMI below 18.5 as major predictors, with an AUROC of 0.661 [8].

However, most of these studies were based on Western health care systems and patient characteristics, limiting their direct application in domestic settings, particularly in medically underserved areas with unique health care environments. The health care system structure, insurance systems, patient health care utilization patterns, disease patterns, and geographical accessibility vary significantly by country and region. Moreover, social determinants of health, such as socioeconomic status, education level, and geographic location, have been shown to account for substantial portions of predictive power in electronic health records–based research [9], further emphasizing the need for locally adapted prediction models. However, high predictive performance alone is insufficient for clinical implementation. For health care providers to trust and use the model in real-world settings, a clear interpretation of how the model performs predictions and which factors increase revisit risk is essential. Tertiary hospitals in medically underserved areas require prediction models that can efficiently use limited medical resources, provide customized discharge plans that consider patients’ geographical accessibility constraints, and establish effective linkages with regional health care institutions. However, research on systematic revisit prediction models that reflect the characteristics of medically underserved domestic areas remains insufficient.

Therefore, this formative study aimed to develop and validate an ML-based model for predicting 30-day ED revisits using EMR data from a tertiary hospital serving a medically underserved area. The 30-day time frame was selected for 3 reasons: (1) it is the most widely adopted window in hospital readmission and ED revisit literature, facilitating direct benchmarking against prior studies; (2) it captures not only early clinical deterioration but also failures in postdischarge management and primary care follow-up, which are particularly relevant in medically underserved areas where timely outpatient access is limited; and (3) it aligns with quality indicator frameworks used by the Health Insurance Review and Assessment Service (HIRA) in South Korea. To our knowledge, this is the first study to apply XGBoost with SHAP-based interpretability to an unselected adult ED population in a medically underserved Korean regional center, addressing a population with high rural residency (12,681/36,230, 35%), advanced aging, and limited primary care infrastructure, which are not represented in prior Asian ML-based ED revisit prediction literature [8]. As a proof-of-concept study, this research establishes the feasibility of implementing ML-based prediction systems in resource-limited settings and provides methodological foundations for future multicenter validation studies.

## Methods

### Study Design and Population

This study is reported in accordance with the TRIPOD (Transparent Reporting of a Multivariable Prediction Model for Individual Prognosis or Diagnosis) guidelines; a completed TRIPOD checklist is provided in Checklist 1. This single-center retrospective cohort study was conducted at Gangneung Asan Hospital. The study period was set from January 1 to December 31, 2023, to sufficiently reflect seasonal variations and ensure an adequate sample size for stable model development. Gangneung Asan Hospital is a regional tertiary hospital in the eastern region of Gangwon Province, South Korea, and a 700-bed tertiary care institution. The ED is designated as a regional emergency medical center with approximately 50,000 annual patient visits and is staffed 24 hours a day by board-certified emergency medicine specialists [10]. The inclusion criteria were adult patients aged 18 years and over who visited the ED of Gangneung Asan Hospital during the study period and were discharged home after ED treatment. Patients who died in the ED, were transferred to other hospitals, were admitted, were left against medical advice, or had incomplete medical records were excluded.

### Data Collection and Variable Definition

All data were extracted from the EMR system of the Gangneung Asan Hospital. The variables were selected by comprehensively considering the major predictors identified in previous studies. Demographic characteristics included sex, age, and residential area. Visit-related variables included the day of visit, time of visit, route of arrival, and mode of transportation. Vital signs, as objective indicators immediately available in the ED, included blood pressure, pulse rate, respiratory rate, body temperature, and oxygen saturation. Clinical characteristics included consciousness level, pain score, and KTAS (Korean Triage and Acuity Scale) score. The tests and procedures included blood and urine tests, electrocardiography, plain radiography, computed tomography (CT), magnetic resonance imaging, ultrasonography, contrast studies, endoscopy, surgery, antibiotics, analgesics, vasopressors, anticoagulants, and fluid therapy. Comorbidities included liver disease, hypertension, diabetes, heart disease, kidney disease, respiratory disease, surgical history, cerebrovascular disease, and malignancy, thereby reflecting the importance of multimorbidity management. The primary outcome variable, 30-day ED revisit, was defined as an unplanned return to the same ED within 30 days of the initial visit. Variable selection and feature engineering were performed only on the training dataset to prevent outcome variable leakage, and the test set was used solely for model performance evaluation. Data preprocessing, variable transformation, and class imbalance correction were performed consistently according to predefined protocols.

### Data Preprocessing and Splitting

For missing values, continuous variables were imputed with median values and categorical variables with mode values. Outliers in vital signs with clinically impossible values were removed. Categorical variables were processed using one-hot encoding, and continuous variables were standardized using the StandardScaler. The entire dataset was split into training (50%), validation (30%), and test (20%) datasets using stratified sampling. Owing to the severe class imbalance with a revisit rate of 2.2% (798/36,230), different class-imbalance correction strategies were applied based on the model type. For XGBoost, the built-in scale_pos_weight parameter was used to up-weight the minority class (ratio of negative to positive cases, approximately 44:1). SMOTE (Synthetic Minority Over-sampling Technique) was applied exclusively to the training data for ElasticNet and random forest, as these models lack an equivalent native class-weight parameter. SMOTE was applied only to the training set; the validation and test sets were left unmodified to prevent data leakage. These methods were not combined for any single model to avoid double correction.

Missing data rates were less than 5% for all key variables. Outlier removal was based on predefined clinical thresholds (systolic BP: <60 or >250 mm Hg; diastolic BP: <40 or >150 mm Hg; heart rate: <30 or >200 bpm; respiratory rate: <8 or >40 breaths per minute; oxygen saturation: <50% or >100%; and body temperature: <34 °C or >42 °C).

### Prediction Model Development

Three ML models were selected to balance interpretability and predictive performance: ElasticNet logistic regression provides a baseline linear model with feature selection capability, random forest offers ensemble learning with inherent interpretability through feature importance, and XGBoost represents state-of-the-art gradient boosting for maximum predictive power. Three models were developed and compared. First, ElasticNet logistic regression, combining L1 and L2 regularization, was configured with l1_ratio=0.5. Second, XGBoost was configured with n_estimators=500, learning_rate=0.05, and max_depth=5, and the class imbalance was adjusted using scale_pos_weight. Third, random forest was configured with n_estimators=300, max_depth=10, and class_weight=“balanced.” The hyperparameters were optimized using 5-fold cross-validation and a grid search.

### Model Evaluation and Interpretation

Model performance was evaluated using AUROC, sensitivity, specificity, precision, and *F*_1_-score, with 95% CI calculated through bootstrap resampling. Calibration was assessed using calibration curves and the Brier score for all 3 models. To benchmark the incremental value of ML, a standard logistic regression model without regularization (penalty=none, SMOTE-balanced) was additionally trained on the same feature set and data split. SHAP analysis was performed for model interpretation to visualize the global importance of each variable and local contributions to individual predictions. SHAP dependence plots were used to identify nonlinear relationships and thresholds for each variable. To assess the stability of SHAP-derived variable importance rankings, the XGBoost model was retrained across 5 independent random seeds (42, 123, 456, 789, and 1024), and rank consistency was evaluated using rank SD. A prespecified stability criterion of SD≤2 was applied. Performance metrics at various thresholds were compared, and optimal cutoff points were determined by considering clinical utility.

### Statistical Analysis and Sample Size Calculation

All analyses were performed using Python 3.9, with scikit-learn 1.0.2, XGBoost 1.6.1, and SHAP 0.41.0. Statistical significance was set at *P*<.05. Bootstrap resampling (n=1000) was used to calculate 95% CI for the performance metrics. Categorical variables were compared using *χ*^2^ tests, and continuous variables were compared using Mann-Whitney *U* tests for nonparametric data and *t* tests for parametric data. Based on the expected revisit rate of 2% to 3% and a desired sensitivity of 0.90, the minimum required sample size was calculated as 30,000 patients to achieve adequate statistical power (80%) with *α*=.05, using the pwr package in R.

### Ethical Considerations

This study was approved by the institutional review board (IRB) of Gangneung Asan Hospital (IRB GNAH 2025-06-001). The requirement for informed consent was waived by the IRB due to the retrospective nature of the study and the use of deidentified data.

## Results

### Baseline Characteristics

During the study period, 48,567 patients visited the ED, and 36,230 met the inclusion criteria for the final analysis. Among all patients, 798 (2.2%) revisited within 30 days. Table 1 shows a comparison of the characteristics of the revisit and nonrevisit groups.

**Table 1.** Baseline characteristics of study population.^a^

Characteristics	Total (N=36,230)	Nonrevisit (n=35,432)	Revisit (n=798)	*P* value
Demographics				
Age (years), mean (SD)	54.3 (19.2)	54.1 (19.2)	62.1 (17.8)	<.001
Male, n (%)	18,476 (51)	18,065 (51)	411 (51.5)	.77
Rural residence, n (%)	12,681 (35)	12,372 (34.9)	309 (38.7)	.03
Visit characteristics, n (%)				
Ambulance use	7246 (20)	7013 (19.8)	233 (29.2)	<.001
KTAS^b^ 1‐2 (severe)	4100 (11.3)	3953 (11.2)	147 (18.4)	<.001
Vital signs, median (IQR)				
Oxygen saturation (%)	98 (96‐99)	98 (96‐99)	96 (93‐98)	<.001
Systolic BP^c^ (mmHg)	130 (118‐145)	130 (118‐145)	125 (110‐142)	<.001
Comorbidities, n (%)				
Liver disease	1326 (3.7)	1205 (3.4)	121 (15.2)	<.001
Heart disease	4515 (12.5)	4287 (12.1)	228 (28.6)	<.001
Diabetes	6702 (18.5)	6416 (18.1)	286 (35.8)	<.001
Tests and procedures				
CT^d^ performed, n (%)	10,508 (29)	10,147 (28.6)	361 (45.2)	<.001
Antibiotic administered, n (%)	7857 (21.7)	7548 (21.3)	309 (38.7)	<.001
Length of stay, h, median (IQR)	2.9 (1.8‐4.7)	2.8 (1.8‐4.6)	4.2 (2.7‐6.8)	<.001

a Primary diagnoses were categorized into major disease groups but are not shown due to space constraints. The most common diagnostic categories were cardiovascular (6594/36,230, 18.2%), gastrointestinal (5688/36,230, 15.7%), and respiratory (4855/36,230, 13.4%) conditions. Comparison of demographic, clinical, and visit characteristics between patients who revisited the emergency department within 30 days and those who did not. Continuous variables are presented as mean (SD) or median (IQR) and categorical variables as n (%). Statistical significance was determined using the appropriate tests for each variable type.

b KTAS: Korean Triage and Acuity Scale.

c BP: blood pressure.

d CT: computed tomography.

The mean age of the revisit group was 62.1 (SD 17.8) years, significantly higher than 54.1 (SD 19.2) years in the nonrevisit group (*P*<.001). Ambulance utilization was higher in the revisit group (233/798, 29.2% vs 7013/35,432, 19.8%; *P*<.001), and the proportion of severe patients (KTAS 1‐2) was also higher in the revisit group (147/798, 18.4% vs 3953/35,432, 11.2%). Among the comorbidities, the prevalence of liver disease (121/798, 15.2% vs 1205/35,432, 3.4%), heart disease (228/798, 28.6% vs 4287/35,432, 12.1%), and diabetes (286/798, 35.8% vs 6416/35,432, 18.1%) was significantly higher in the revisit group (all *P*<.001). The ED length of stay was significantly longer in the revisit group, with a median of 4.2 (IQR 2.7‐6.8) hours compared to 2.8 (IQR 1.8-4.6) hours in the nonrevisit group (*P*<.001).

### Model Performance Comparison

The test dataset performances of the 3 models are presented in Table 2. XGBoost showed the best performance with an AUROC of 0.90 (95% CI 0.88‐0.92), achieving a sensitivity of 0.94 and a specificity of 0.69 at a cutoff rate of 0.10, correctly identifying 94% (750/798) of revisit patients. ElasticNet logistic regression showed the second-best performance, with an AUROC of 0.89, and random forest recorded an AUROC of 0.87.

**Figure 1.**
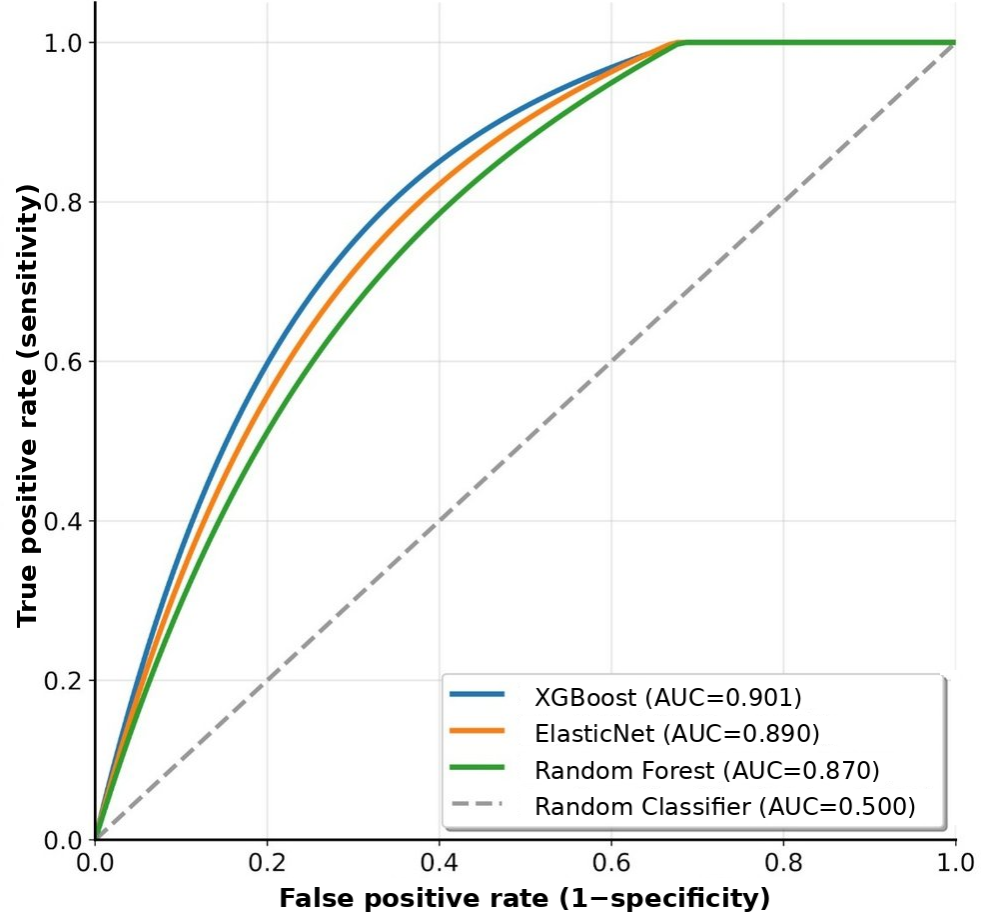
Receiver operating characteristic (ROC) curves for machine learning models. ROC curves compare the performance of the 3 machine learning models for predicting 30-day emergency department revisits. Extreme gradient boosting (XGBoost) achieved the highest area under the curve (AUC) of 0.901, followed by ElasticNet logistic regression (AUC 0.890) and random forest (AUC 0.870). The diagonal dashed line represents the random classifier performance (AUC 0.500).

Figure 1 shows a comparison of the ROC curves for the 3 models.

XGBoost showed the best performance, with an AUROC of 0.901, representing top-tier predictive power compared to previous studies.

**Table 2.** Comparison of model predictive performance^a^.

Model	AUROC^b^ (95% CI)	Sensitivity^c^	Specificity^c^	Precision^c^	*F*_1_-score^c^	Cutoff
XGBoost^d^	0.90 (0.88‐0.92)	0.94	0.69	0.09	0.16	0.10
ElasticNet	0.89 (0.87‐0.91)	0.79	0.82	0.12	0.21	0.20
Random forest	0.87 (0.85‐0.89)	0.83	0.78	0.10	0.18	0.10

a Performance metrics for the 3 machine learning models on the test dataset were evaluated. All metrics were calculated at the optimal cutoff threshold for each model, which maximizes the balance between sensitivity and specificity. CIs were calculated using bootstrap resampling (n=1000).

b AUROC: area under the receiver operating characteristic.

c Measured at the optimal cutoff for each model.

d XGBoost: extreme gradient boosting.

### SHAP Analysis Results

**Figure 2.**
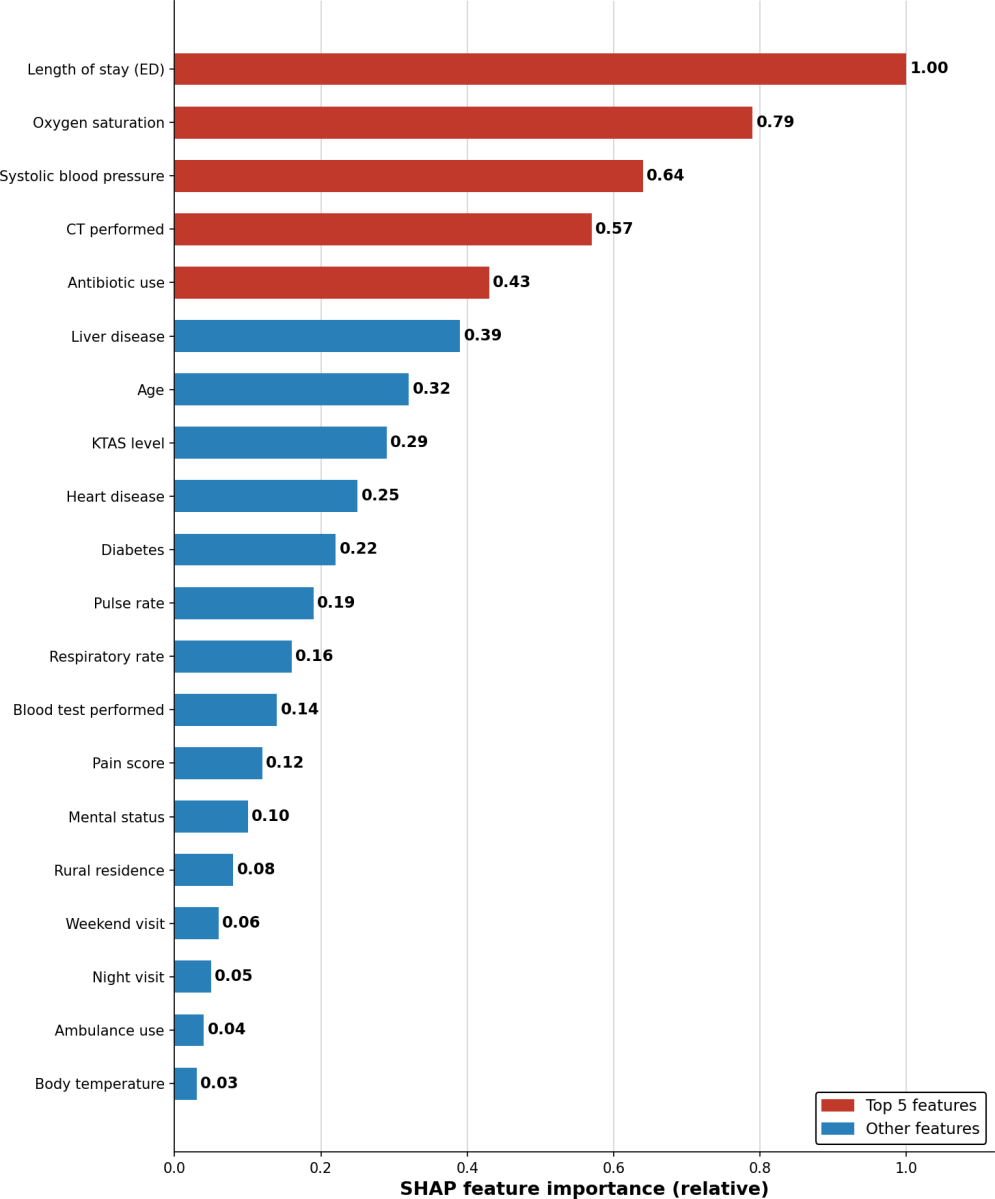
Shapley additive explanations (SHAP) feature importance analysis. SHAP feature importance ranking for the extreme gradient boosting (XGBoost) model. The horizontal bar chart shows the relative importance of the top predictive variables, with emergency department (ED) length of stay being the most important predictor (relative importance: 1.00), followed by oxygen saturation (0.79) and systolic blood pressure (0.64). Red bars indicate the top 5 most important features. CT: computed tomography; KTAS: Korean Triage and Acuity Scale.

The SHAP analysis of the XGBoost model identified the most important variables for revisit prediction (Table 3, Figure 2).

**Table 3.** SHAP^a^ analysis results—Major predictive variables (top 8)^b^.

Rank	Variable	Relative importance	Clinical significance
1	ED^c^ length of stay	1.00	Proxy indicator of patient severity and complexity
2	Oxygen saturation	0.79	Physiological instability, respiratory dysfunction
3	Systolic blood pressure	0.64	Cardiovascular stability, shock state
4	CT^d^ performed	0.57	Indirect indicator of disease severity
5	Antibiotic use	0.43	Presence of infectious disease
6	Liver disease	0.39	Major comorbidity, poor prognosis
7	Age	0.32	Decreased physiological reserve
8	KTAS^e^ level	0.29	ED severity classification

a SHAP: Shapley additive explanations.

b Top 8 predictive variables ranked by SHAP importance values from the extreme gradient boosting model are presented. Relative importance was normalized, with the most important variable (ED length of stay) set to 1.00. Clinical significance describes the potential mechanism by which each variable contributes to revisit risk.

c ED: emergency department.

d CT: computed tomography.

e KTAS: Korean Triage and Acuity Scale.

ED length of stay emerged as the most important predictive variable, followed by oxygen saturation, systolic blood pressure, CT scan performed, antibiotic use, liver disease, age, and KTAS level in order of importance. Binary variables, including CT scan performed (fourth), antibiotic use (fifth), and liver disease (sixth), also showed high SHAP importance, with the revisit risk significantly increasing when these variables were present.

**Figure 3.**
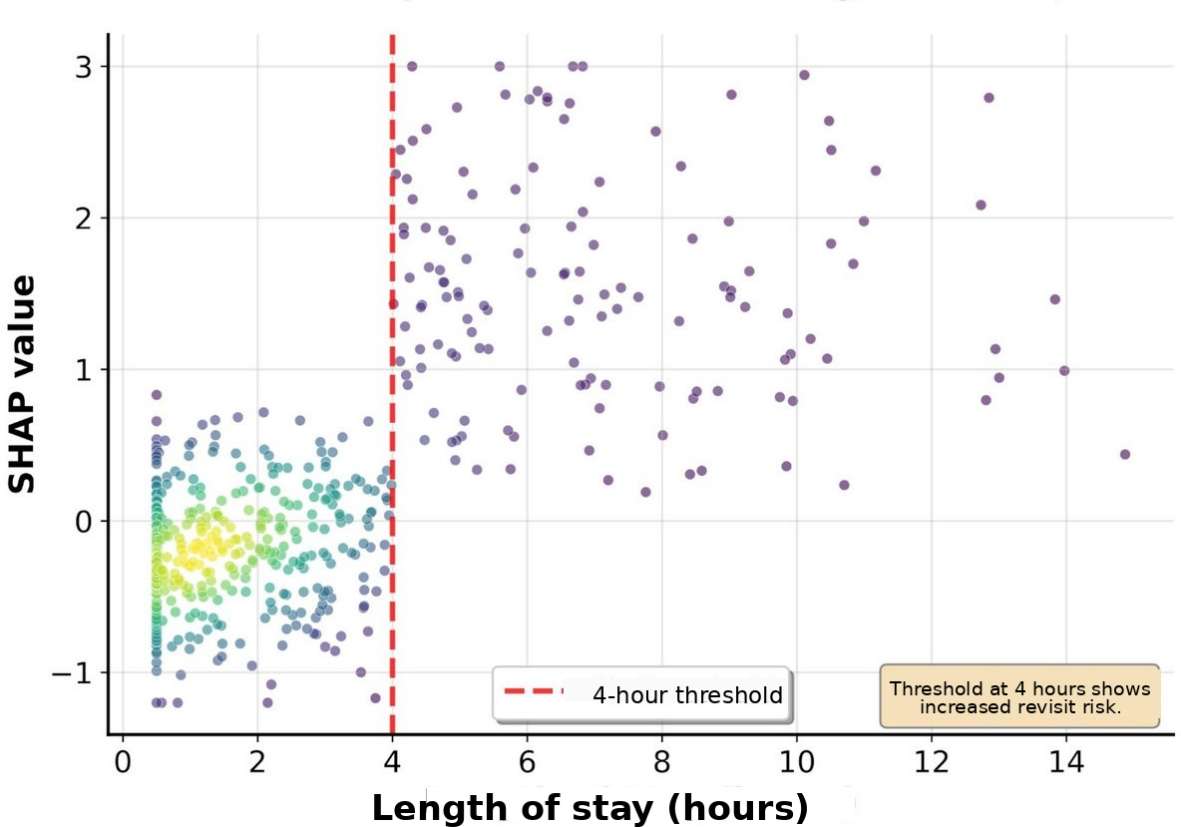
SHAP dependence plot for ED length of stay. The relationship between ED length of stay and SHAP values shows an increased revisit risk beyond the 4-hour threshold. Each point represents an individual patient, with colors indicating the feature interaction effects. The red dashed line marks the 4-hour threshold, where the revisit risk significantly increases*.* ED: emergency department; SHAP: Shapley additive explanations.

**Figure 4.**
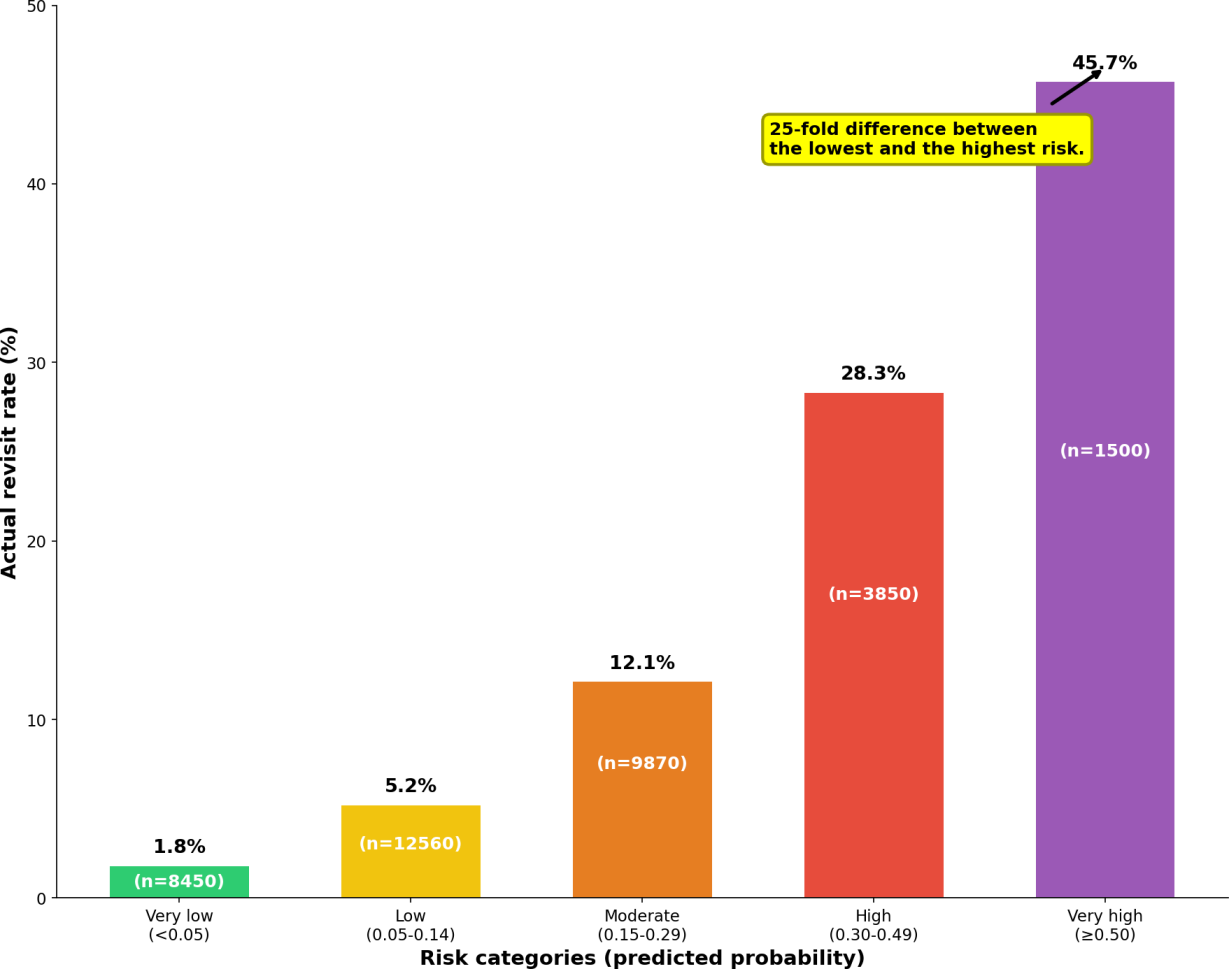
Risk stratification and actual revisit rates. The 5-tier risk classification showed actual 30-day revisit rates ranging from 1.8% (very low risk) to 45.7% (very high risk), demonstrating a 25-fold difference between the risk categories. The sample sizes for each risk category are shown in parentheses.

**Figure 5.**
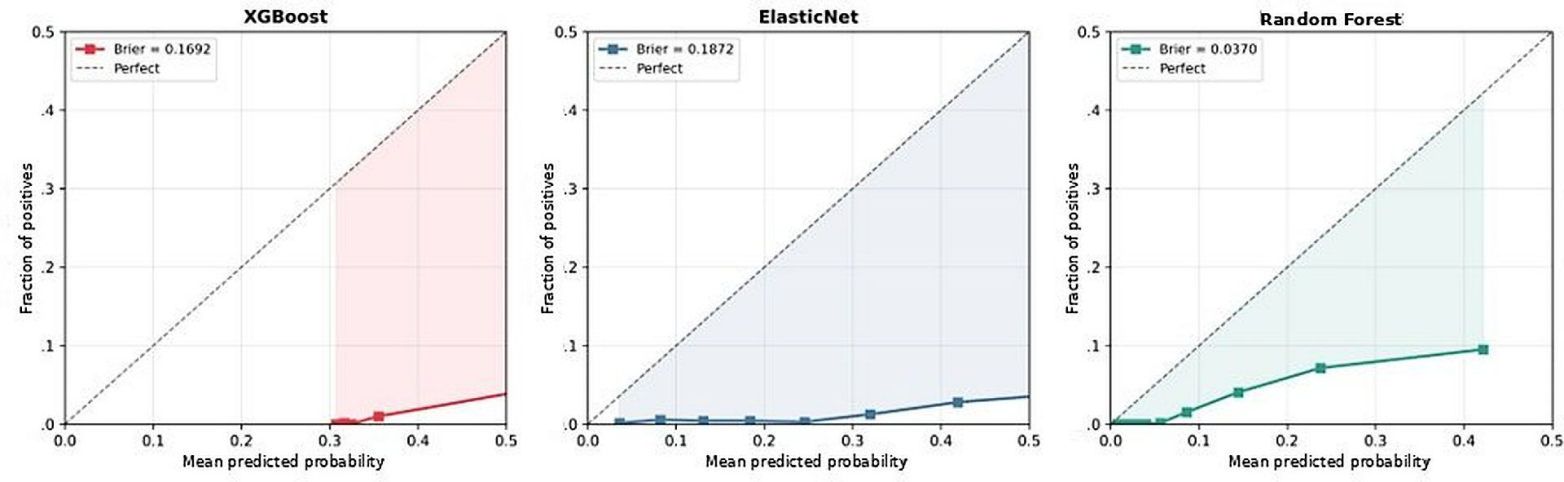
Calibration curves for the 3 machine learning models. The calibration curves show the relationship between the mean predicted probability and the fraction of positives for each model. Brier scores were 0.169 (XGBoost), 0.187 (ElasticNet), and 0.037 (random forest). Both XGBoost and ElasticNet showed overestimation of risk probabilities, consistent with the effects of SMOTE oversampling at low event prevalence. The dashed diagonal line represents perfect calibration. SMOTE: Synthetic Minority Over-sampling Technique; XGBoost: extreme gradient boosting.

**Figure 6.**
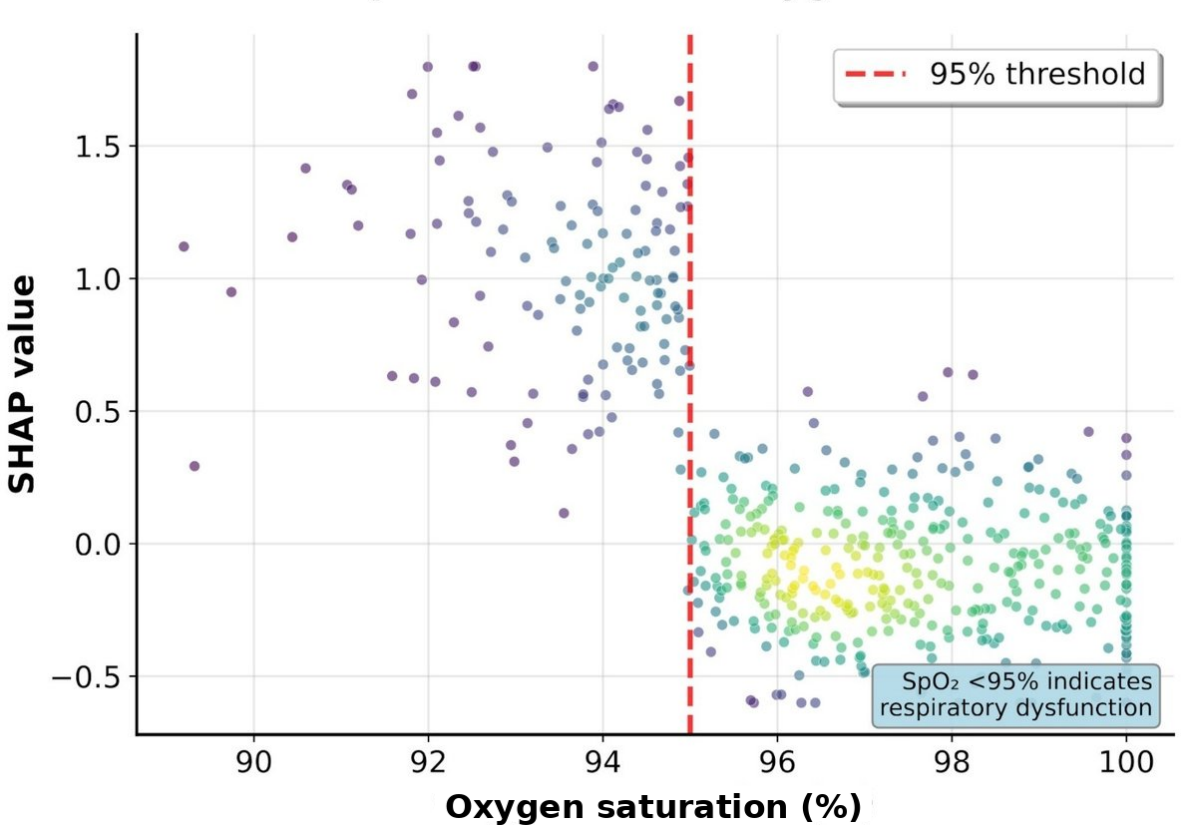
SHAP dependence plot for oxygen saturation. Nonlinear relationship between oxygen saturation and revisit risk. Patients with SpO_2_<95% showed markedly increased SHAP values, indicating that respiratory dysfunction is a strong predictor. The dashed red line marks the 95% threshold. SHAP: Shapley additive explanations.

The SHAP dependence plot analysis focused on 4 continuous variables with particularly important nonlinear relationships: ED length of stay, oxygen saturation, age, and systolic blood pressure (Figures 3-6).

The ED length of stay showed an overall trend of increasing revisit risk with longer stays, with particularly high SHAP values observed in patients staying for more than 4 hours, although with considerable variability. Oxygen saturation showed an increased revisit risk at lower values, with particularly high SHAP values observed in some patients (<95%). Age showed an overall trend of increasing SHAP values with advancing age, and systolic blood pressure showed a U-shaped pattern with increased revisit risk in both the hypotensive and hypertensive ranges.

### Threshold Optimization and Risk Stratification

**Figure 7.**
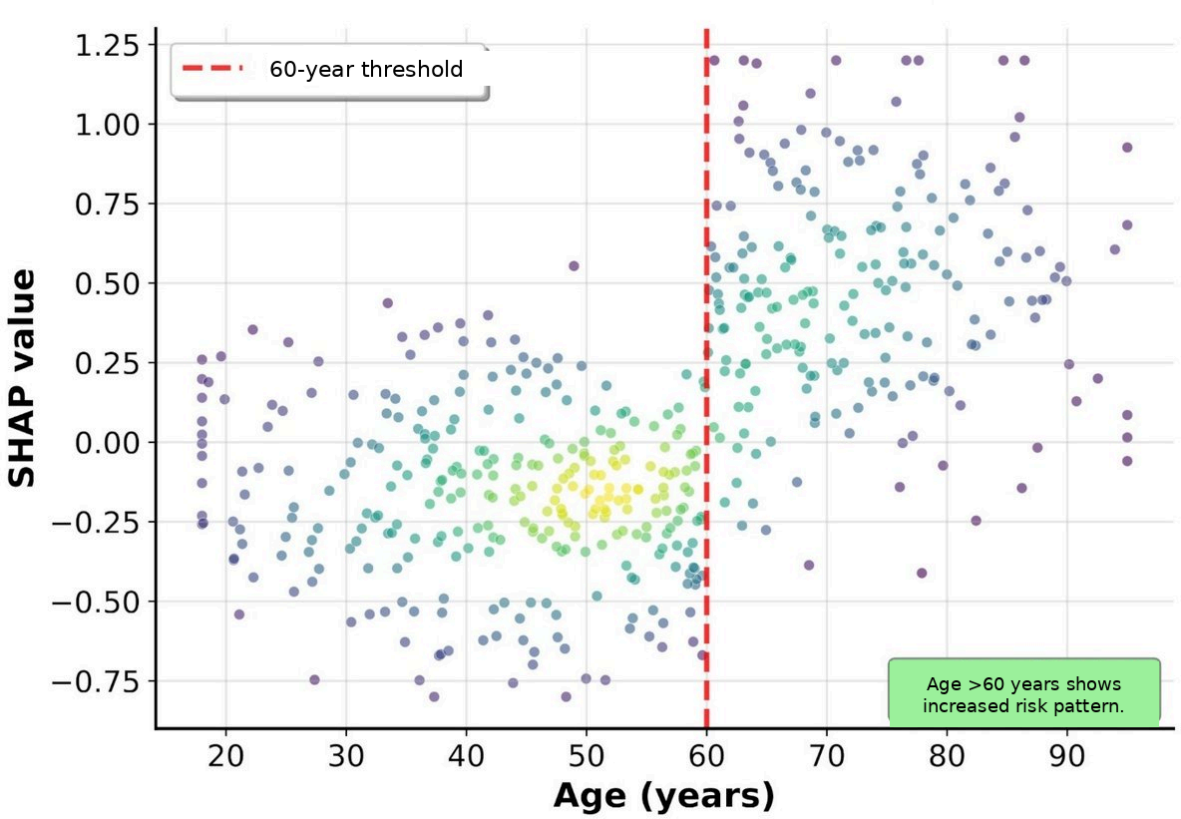
SHAP dependence plot for age. Age-related patterns in revisit prediction showed an increased risk for patients older than 60 years. The scatter plot demonstrates variable individual risks despite similar ages. The dashed red line marks the 60-year threshold. SHAP: Shapley additive explanations.

To validate the clinical utility of the developed model, 5-tier risk stratification showed actual revisit rates ranging from 1.8% (152/8450) in the very low-risk group to 45.7% (686/1500) in the very high-risk group, representing a more than 25-fold difference in risk (Figure 7).

This risk stratification can be utilized in clinical practice as follows: the very high-risk group (≥0.50, actual revisit rate n=1500, 45.7%) should consider delayed discharge with telephone confirmation within 48 hours; the high-risk group (0.30‐0.49, n=3850, 28.3%) is recommended for discharge coordinator assignment and early outpatient appointments; the moderate-risk group (0.15‐0.29, n=9870, 12.1%) requires telephone follow-up within 7 days and enhanced education; the low-risk group (0.05‐0.14, n=12,560, 5.2%) requires standard discharge education; and the very low-risk group (<0.05, n=8450, 1.8%) can be managed with minimal intervention.

### Calibration Analysis and Model Benchmarking

Calibration analysis demonstrated Brier scores of 0.169, 0.187, and 0.037 for XGBoost, ElasticNet, and random forest, respectively (Figure 5). The calibration curves revealed a tendency for overestimation of predicted probabilities in XGBoost and ElasticNet, which is characteristic of rare-event prediction models trained with SMOTE oversampling at low prevalence (798/36,230, 2.2%). Random forest demonstrated lower absolute predicted probabilities due to SMOTE compressing the probability scale. For clinical deployment, probability recalibration would be recommended before using absolute predicted probabilities for risk communication. Benchmarking against a standard logistic regression model (no regularization, SMOTE-balanced) confirmed the incremental value of the ML approach: standard logistic regression achieved an AUROC of 0.789 (95% CI 0.756‐0.820), compared to XGBoost’s AUROC of 0.874 (95% CI 0.856‐0.890), an incremental gain of +0.085 AUROC points (Figure S1 in Multimedia Appendix 1). SHAP variable importance rankings were highly stable across 5 independent random seeds (seeds 42, 123, 456, 789, and 1024; mean AUROC of 0.871, SD 0.006; range 0.014): the top 2 features maintained identical rank ordering (SD 0.00), and all 10 of the top 10 features satisfied the prespecified stability criterion of rank SD≤2 (Figure S2 in Multimedia Appendix 1).

## Discussion

### Key Findings and Formative Contributions

This formative study successfully developed and validated an ML-based prediction model for 30-day ED revisits in a medically underserved tertiary hospital. The XGBoost model achieved an AUROC of 0.90, demonstrating that high-performance prediction modeling is feasible even in resource-limited settings. Beyond the technical achievements, this research establishes a replicable methodological framework for similar institutions serving underserved populations.

### Performance Comparison and Model Validation

The XGBoost model developed in this study achieved an AUROC of 0.90, representing a top-tier performance compared with previous studies. This performance is particularly meaningful when compared to previous studies in similar clinical environments. Deep learning–based Tree-GloVe embedding and temporal attention models achieved an AUROC of 0.98 on Medical Information Mart for Intensive Care III (MIMIC-III) and an AUROC of 0.85 on the New York Medicaid datasets in controlled research environments [11]. Our achievement in an actual clinical environment demonstrates the practical applicability of our approach. Large-scale studies have confirmed the potential of ML approaches. Research using the National Readmissions Database in the United States developed disease-specific prediction models, achieving an AUROC of 0.876 for diabetes [12], whereas Taiwan-based studies recorded an AUROC of 0.765, confirming that risk factors differ between 14-day and 30-day readmissions [13]. Compared to specialized patient populations, the performance of our model is particularly noteworthy. External validation studies of post-chemotherapy ED visit prediction models showed relatively low performance with an AUROC of 0.65 [14], while studies on lower gastrointestinal bleeding patients achieved an AUROC of 0.83 [15]. Unlike these disease-specific models, our approach successfully predicted revisits across all ED presentations, thereby enhancing its practical utility in real-world emergency settings. To contextualize our patient population within the national landscape, we compared key characteristics with benchmarks from the National Emergency Department Information System (NEDIS) [16]. The mean patient age in our cohort (54.3 y) exceeds the national ED average, consistent with the accelerated aging profile of Gangwon Province. Our rural residency proportion (12,681/36,230, 35%) substantially exceeds the national ED average, reflecting the geographically dispersed catchment area of Gangneung Asan Hospital. The 30-day revisit rate of 2.2% (798/36,230) falls within the reported range of 2% to 5% for comparable Korean regional emergency centers. The diagnostic case mix (6594/36,230, 18.2% cardiovascular, 5688/36,230, 15.7% gastrointestinal, and 4855/36,230, 13.4% respiratory) is broadly comparable to national tertiary ED distributions. We acknowledge that formal benchmarking is limited by the absence of a contemporaneous matched control institution, and multicenter validation remains the next priority step to assess generalizability. To contextualize the incremental value of the ML approach, we benchmarked XGBoost against a standard logistic regression model (no regularization) trained on the same feature set. The standard logistic regression achieved an AUROC of 0.789 (95% CI 0.756‐0.820), compared to XGBoost’s AUROC of 0.874 (95% CI 0.856‐0.890), representing an incremental gain of +0.085 AUROC points (Figure S1 in Multimedia Appendix 1). In the top-decile high-risk group, XGBoost identified patients with substantially higher actual revisit rates than logistic regression, suggesting that the nonlinear interactions captured by gradient boosting—particularly between ED length of stay, KTAS severity, and comorbidity patterns—contributed meaningfully beyond what linear models can capture.

### Clinical Interpretation of Key Predictive Variables

Clinical interpretation of the major predictive variables identified through SHAP analysis revealed that ED length of stay, being the most important predictive variable, is a comprehensive indicator reflecting patient complexity and severity. The SHAP dependence plot analysis showed a trend of increasing revisit risk with longer ED stays, with a particularly high risk observed in patients staying for more than 4 hours. This coincides with the time when most diagnoses and initial treatments are completed in emergency medicine, and patients remaining in the ED beyond this time are likely to have more complex medical problems. A study on 30-day readmission prediction after percutaneous coronary intervention also identified length of stay as a major predictive variable, which is consistent with our findings [17]. A sharp increase in revisit risk when oxygen saturation falls below 95% serves as an objective indicator of respiratory instability, and even mild decreases in oxygen saturation may suggest potential respiratory problems. This finding contrasts with a study on pain-related ED revisits in middle-aged and older patients, which found health literacy to be more important than physiological indicators [18]. Our study’s emphasis on objective physiological indicators may reflect the geographic characteristics of our patient population, as patients in medically underserved areas often delay seeking care until their symptoms become more severe. The U-shaped relationship of blood pressure reflects cardiovascular instability, suggesting problems with blood pressure regulation rather than hypertension or hypotension. The presence of comorbidities, including liver disease, has emerged as a strong predictor of revisits and reflects complex pathophysiological interactions, including decreased overall physiological reserve and abnormal drug metabolism due to liver dysfunction. The high predictive importance of CT performance (fourth) and antibiotic use (fifth) deserves special attention. These binary variables are likely to serve as proxies for clinical decision-making complexity. CT imaging is typically ordered when diagnostic uncertainty exists or when serious pathology is suspected, reflecting cases in which initial clinical assessment alone is insufficient. Similarly, antibiotic administration in the ED suggests acute infectious processes that may not be fully resolved at discharge, potentially leading to clinical deterioration and subsequent revisits. These treatment-related variables capture the implicit clinical judgment of emergency physicians regarding patient acuity and diagnostic complexity, which traditional scoring systems have failed to incorporate.

### Model Characteristics and Risk Stratification System

**Figure 8.**
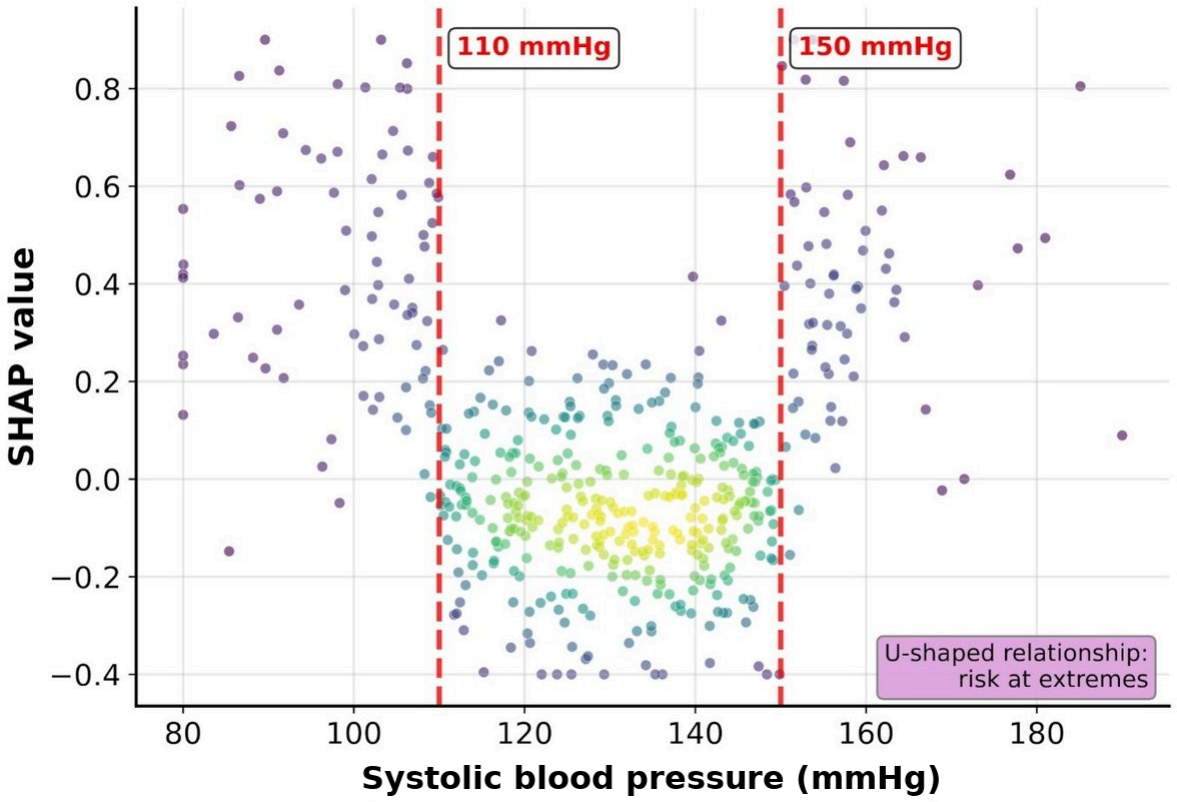
SHAP dependence plot for systolic blood pressure. A U-shaped relationship between blood pressure and revisit risk is observed. Both hypotension (<110 mm Hg) and hypertension (>150 mm Hg) contributed to an increased revisit probability. The red dashed lines mark the 110 and 150 mm Hg thresholds. SHAP: Shapley additive explanations.

The considerable variance in the SHAP dependence plots highlights an important limitation: patients with identical variable values may have different revisit risks. This reflects both the probabilistic nature of prediction models and the influence of unmeasured factors, particularly the social determinants of health that are challenging to capture in routine clinical data. This variability indicates that various patient characteristics and complex socioeconomic factors in medically underserved areas may influence revisits, representing elements that are difficult to capture using clinical indicators alone. All variables showed large interindividual variability, suggesting that these thresholds should be interpreted as reference points that require careful observation during risk assessment rather than absolute criteria. The 5-tier risk stratification system developed in this study provides concrete guidelines that are immediately applicable in practice. A Medicare data-based Bayesian survival model study showed that explainable structure-based models can achieve a performance equivalent to that of black-box models [19], and an ML study on diabetic inpatients achieved an AUROC of 0.661 with random forest using down-sampling [20], and the only comparable Asian ED revisit prediction study achieved an AUROC of 0.661 using a nomogram approach in older Chinese patients [8]. Our XGBoost model demonstrated superior performance while securing high interpretability through SHAP. Comparative studies across different patient populations support our approach: pediatric patient studies reported that the LACE index has different cutoff values from those of adults [21], oncology patient studies confirmed that sufficient prediction is possible with information from the first 48 hours of admission [22], and specialized studies using advanced techniques such as Graph Neural Networks demonstrated effective learning of complex relationship structures [23]. The high sensitivity (0.94) achieved by our XGBoost model results from focusing on patients with high-risk revisits, which is deemed a more appropriate approach from a patient safety perspective. Calibration analysis demonstrated Brier scores of 0.169, 0.187, and 0.037 for XGBoost, ElasticNet, and random forest, respectively (Figure 8). The calibration curves revealed a tendency for overestimation of predicted probabilities in XGBoost and ElasticNet, a pattern characteristic of rare-event prediction models (prevalence 2.2%) trained with SMOTE oversampling. Random forest demonstrated lower absolute predicted probabilities owing to SMOTE compressing the probability scale. For clinical deployment, probability recalibration (eg, Platt scaling or isotonic regression) would be recommended before using absolute predicted probabilities for risk communication. SHAP variable importance rankings were highly stable across 5 independent random seeds (seeds 42, 123, 456, 789, and 1024; mean AUROC 0.871, range 0.014): the top 2 features (ED length of stay and KTAS level) maintained identical rank ordering across all seeds (SD 0.00), and all 10 of the top 10 features satisfied the prespecified stability criterion of rank SD≤2 (Figure S2 in Multimedia Appendix 1).

The thresholds identified through the SHAP analysis align well with established clinical knowledge. The 4-hour ED length of stay threshold corresponds to typical ED process times, after which complex cases requiring extended workup or observation are differentiated from straightforward presentations. The 95% oxygen saturation cutoff matches the clinical definition of hypoxemia, whereas the 60-year age threshold reflects the transition to increased physiological vulnerability. The U-shaped blood pressure relationship with increased risk below 110 mm Hg and above 150 mm Hg corresponds to the established definitions of hypotension and hypertensive urgency, respectively. These data-driven thresholds validate existing clinical intuition while providing quantitative risk stratification.

### Clinical Implementation Strategy

Customized strategies that consider the characteristics of medically underserved areas are important. The significantly higher revisit rate among rural residents in this study reflects limited medical accessibility. Therefore, improving accessibility through the introduction of telemedicine systems and strengthening linkages with local health centers is necessary. To efficiently utilize limited medical resources, they should concentrate on high-risk groups, and protocols for the systematic management of moderate-risk and high-risk patients should be established. For the actual clinical application of the developed model, a phased approach is necessary. During the first 3 months, a pilot system should be built to develop a real-time prediction system linked to the ED EMR, and the user interface should be optimized through health care provider education and feedback collection. Over the following 6 months, the system should be expanded to all ED health care providers while continuously monitoring the prediction accuracy in real time and updating the model’s performance. The early HOSPITAL score study showed that performance equivalent to existing models can be achieved with early admission test results alone [24]. A recent study by Zhang et al [25] demonstrated that acute-phase indicators are particularly important in the ED environment, which is consistent with the immediately available ED variables identified as major predictors in our study.

### Policy Implications and Study Limitations

The expected economic effects of introducing this prediction model include direct benefits such as medical cost savings from reduced revisits, increased operational efficiency from ED overcrowding relief, and a reduction in unnecessary tests and procedures, as well as indirect benefits including improved patient satisfaction, reduced health care provider workload, and improved regional health care quality. Policy considerations should include using it as a quality assessment indicator for EDs in medically underserved areas, reviewing the introduction of prediction model–based reimbursement systems, and supporting the establishment of regionally customized emergency medical systems. The limitations of this study include the need for external validity verification as a single-center study and the possible underestimation of actual revisit rates due to the absence of information on revisits to other hospitals. Additionally, the insufficient inclusion of socioeconomic variables and the inability to utilize unstructured data, such as medical record text, are notable limitations. Using only 1 year of data, failing to adequately reflect long-term temporal patterns is also a limitation. Patterns identified in the SHAP dependence plot analysis showed considerable interindividual variability, suggesting that revisit prediction requires a multivariate approach rather than single-variable thresholds alone.

As a formative, single-center study, several limitations should be acknowledged. First, the 30-day time frame was selected because it is the most widely adopted window in hospital readmission and ED revisit literature, facilitating direct benchmarking, and it aligns with quality indicator frameworks used by the HIRA in South Korea. Nevertheless, this window may not capture all clinically relevant early postdischarge deterioration. Future studies should explore multiple prediction time frames (3, 7, and 30 days) to identify optimal intervention targets, particularly for early postdischarge deterioration. Second, the retrospective definition of “unplanned” revisits may include some subjectivity, though we used systematic criteria based on triage acuity and chief complaints. Third, external validation in other medically underserved settings is necessary to assess generalizability. Fourth, the low revisit prevalence (2.2%) resulted in low precision (XGBoost: 0.09 at the 0.10 threshold), meaning approximately 11 patients must be flagged for each true revisit detected. We argue this tradeoff is acceptable in our resource-limited context where interventions triggered are low cost (telephone follow-up, enhanced discharge education), and the 5-tier risk stratification enables proportional resource deployment. Future implementation studies should formally evaluate the cost-effectiveness of this precision-recall tradeoff using decision curve analysis. Fifth, the model was trained on 2023 data and should be subject to periodic revalidation every 6 to 12 months, with recalibration triggered if the AUROC on prospective validation falls below 0.85 or if the observed revisit rate shifts by more than 1 percentage point. Post-COVID-19 effects on ED utilization patterns in Gangwon Province—including changes in infectious disease burden, deferred chronic disease management, and altered health care–seeking behavior following the 2024 medical-government conflict—represent plausible sources of distributional shift that future monitoring should specifically assess. Despite these limitations, this proof-of-concept study demonstrates that sophisticated ML approaches can be successfully implemented in resource-limited settings.

### Future Research Directions

Future research should include external validation studies in other EDs to assess model generalizability beyond our institution. The significantly higher revisit rate among rural residents observed in this study (309/798, 38.7% vs 12,372/35,432, 34.9%; *P*=.03) reflects geographic access barriers and underscores the need to systematically capture social determinants of health in future models. In South Korea, linkage of hospital EHR data with National Health Insurance Service (NHIS) claims data could provide enriched social determinants of health variables—including income decile, drive time to the nearest clinic, and health literacy proxies—at scale and would enable analysis of factors such as outpatient utilization frequency, medication adherence, and chronic disease management continuity, all of which are potentially relevant to ED revisit risk in medically underserved populations. The application of advanced artificial intelligence techniques, such as natural language processing for unstructured medical record analysis and time-series modeling, could potentially enhance prediction accuracy. Recent advances in natural language processing have shown promise in enhancing prediction models. For instance, Lineback et al [26] demonstrated improved stroke readmission prediction by incorporating unstructured clinical notes, suggesting a valuable avenue for enhancing our model through the integration of free-text emergency physician documentation and nursing notes. Randomized controlled studies would be valuable to verify the actual clinical effectiveness of model-based interventions in reducing revisit rates and improving patient outcomes. This study is particularly significant as it is one of the first systematic revisit prediction studies considering the characteristics of medically underserved areas, suggesting the possibility of establishing regionally customized emergency medical systems that could serve as a model for similar health care environments globally.

## Supplementary material

10.2196/87289 Multimedia Appendix 1 Receiver operating characteristic curves comparing extreme gradient boosting and standard logistic regression (Figure S1) and Shapley Additive explanations variable importance stability across five random seeds (Figure S2).

10.2196/87289 Checklist 1 TRIPOD checklist.

## Abbreviations

AUROC: area under the receiver operating characteristic
CT: computed tomography
ED: emergency department
EMR: electronic medical record
HIRA: Health Insurance Review and Assessment Service
IRB: institutional review board
KTAS: Korean Triage and Acuity Scale
MIMIC-III: Medical Information Mart for Intensive Care III
ML: machine learning
NEDIS: National Emergency Department Information System
NHIS: National Health Insurance Service
SHAP: Shapley additive explanations
SMOTE: Synthetic Minority Over-sampling Technique
TRIPOD: Transparent Reporting of a Multivariable Prediction Model for Individual Prognosis or Diagnosis
XGBoost: extreme gradient boosting
