# Supplementary material for: Machine Learning–Based Prediction Model for 30-Day Emergency Department Revisits in a Medically Underserved Tertiary Hospital: Formative Retrospective Cohort Study
Source: JMIR Form Res. 2026 May 29;10:e87289. doi: 10.2196/87289 (PMC13220723; doi:10.2196/87289)
Supplement: Multimedia Appendix 1 [file formative-v10-e87289-s001.docx]

**Supplementary Figures**


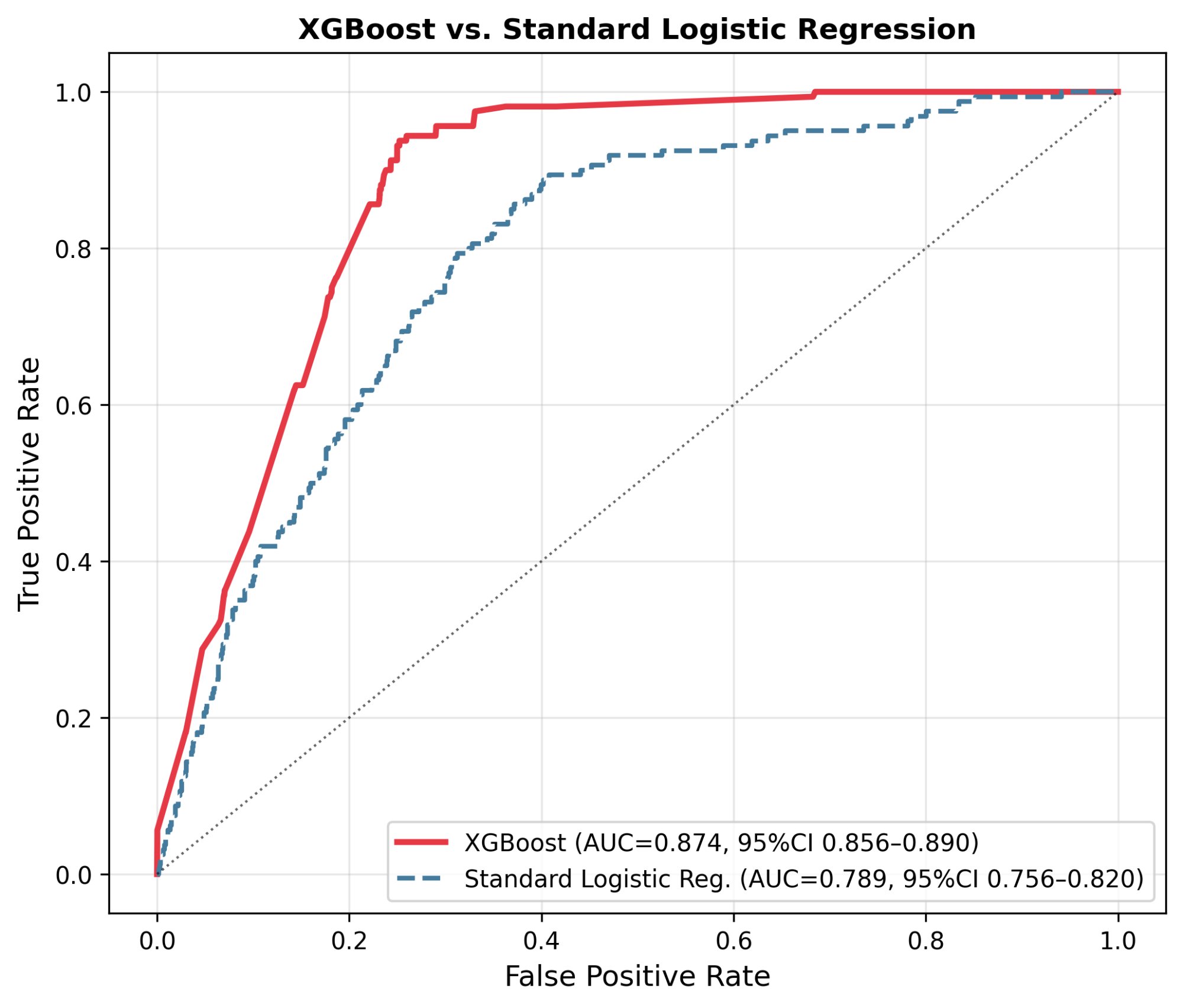


**Supplementary Figure S1. ROC curves comparing XGBoost and standard logistic regression.** *XGBoost (AUC=0.874, 95% CI: 0.856–0.890) outperformed standard logistic regression without regularization (AUC=0.789, 95% CI: 0.756–0.820), representing an incremental gain of +0.085 AUROC points. The larger gap compared to typical EHR-based studies suggests that nonlinear interactions captured by gradient boosting contribute meaningfully in this clinical setting.*


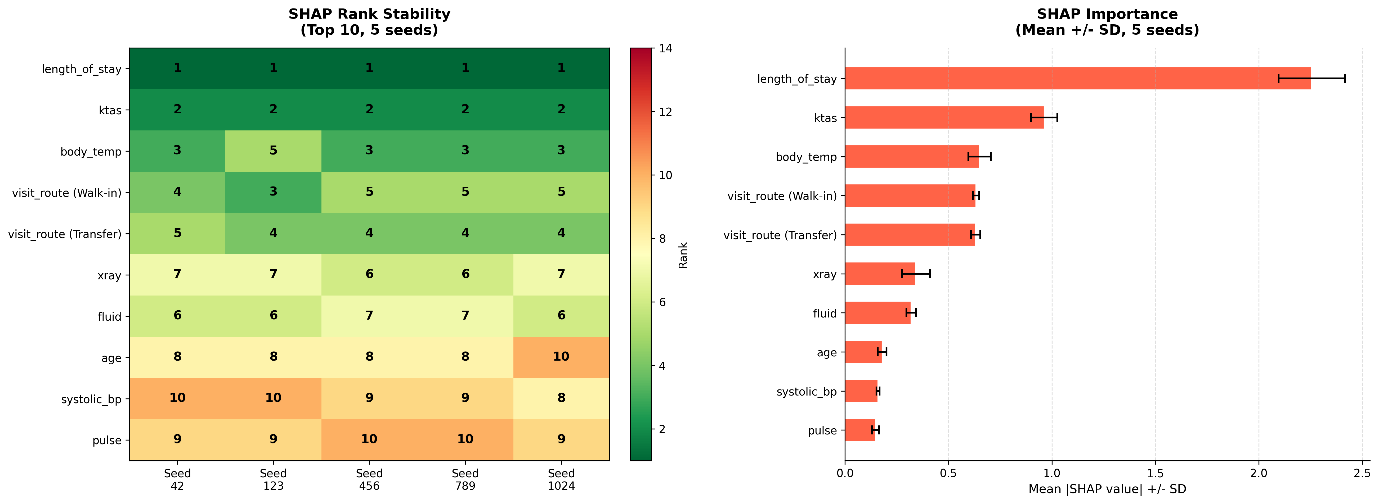


**Supplementary Figure S2. SHAP variable importance stability across five random seeds.** (Left) Heatmap showing SHAP importance ranks for the top 10 features across seeds 42, 123, 456, 789, and 1024. Green indicates lower rank number (higher importance). (Right) Bar chart showing mean ± SD of absolute SHAP values across the five seeds. The top two features (ED length of stay and KTAS level) maintained identical rankings across all seeds (rank SD = 0.00). Features ranked 3rd through 10th (body temperature, visit route, X-ray, fluid administration, age, systolic blood pressure, and pulse rate) showed minor rank fluctuations (rank SD ≤ 0.89). All 10 of the top 10 features satisfied the pre-specified stability criterion of rank SD ≤ 2.
