# Supplementary material for: Machine Learning–Based Prediction Model for 30-Day Emergency Department Revisits in a Medically Underserved Tertiary Hospital: Formative Retrospective Cohort Study
Source: JMIR Form Res. 2026 May 29;10:e87289. doi: 10.2196/87289 (PMC13220723; doi:10.2196/87289)
Supplement: Checklist 1 [file formative-v10-e87289-s002.pdf]

TRIPOD Checklist — Transparent Reporting of a Multivariable Prediction Model for Individual Prognosis or Diagnosis

Manuscript #87289 | JMIR Formative Research

"Machine Learning-Based Prediction Model for 30-Day Emergency Department Revisits in a Medically Underserved Tertiary Hospital"

Item	Section	Page	Where Reported / Comment	✓
**TITLE AND ABSTRACT**				
1. Title	Title	1	Title includes study design (retrospective cohort) and identifies it as a prediction model development study.	✓
2. Abstract	Abstract	1	Structured abstract includes background, methods (cohort, n=36,230, three ML models, SHAP), results (AUROC 0.90, sensitivity 0.94), and conclusions.	✓
**INTRODUCTION**				
3a. Background	Introduction	2–3	Clinical problem (ED revisits as quality indicator), limitations of LACE/HOSPITAL scores, and rationale for ML approach described.	✓
3b. Objectives	Introduction	3	Objective explicitly stated: to develop and validate an ML-based prediction model for 30-day ED revisits in a medically underserved tertiary hospital.	✓
**METHODS — Data**				
4a. Source of data	Methods: Study Design	3–4	Single-center retrospective cohort; Gangneung Asan Hospital ED; January–December 2023; electronic medical records.	✓
4b. Dates	Methods: Study Design	3	Study period: January 1 to December 31, 2023.	✓
5a. Eligibility criteria	Methods: Study Design	3–4	Adults ≥18 years discharged from ED. Exclusions: death in ED, transfer, admission, LAMA, incomplete records.	✓
5b. Setting	Methods: Study Design	3	Regional emergency medical center, 700-bed tertiary hospital, ~50,000 annual ED visits, 24-hour specialist coverage, medically underserved area.	✓
6. Outcome	Methods: Variable Definition	4	30-day unplanned ED revisit defined as return to the same ED within 30 days. Prevalence: 2.2% (798/36,230).	✓
7. Predictors	Methods: Variable Definition	4	Demographics, visit characteristics, vital signs, KTAS, comorbidities, tests, procedures, discharge status, medications — comprehensive EHR-based variables.	✓

Item	Section	Page	Where Reported / Comment	✓
8. Sample size	Methods: Statistical Analysis	6	Minimum 30,000 patients required (expected 2–3% revisit rate, sensitivity 0.90, power 80%, α=0.05); 36,230 enrolled.	✓
9. Missing data	Methods: Data Preprocessing	4–5	Missing data <5% for all key variables; continuous imputed with median, categorical with mode.	✓
**METHODS — Model Development**				
10a. Model building	Methods: Model Development	5	Three models: ElasticNet (L1/L2, l1_ratio=0.5), XGBoost (n_estimators=500, lr=0.05, max_depth=5), Random Forest (n_estimators=300, max_depth=10). Hyperparameters optimized via 5-fold CV + grid search.	✓
10b. Internal validation	Methods: Model Evaluation	5–6	Stratified 50/30/20 split (train/validation/test). Bootstrap resampling (n=1,000) for 95% CIs.	✓
10c. Model specification	Methods: Model Development	5	All model parameters specified. Preprocessing: one-hot encoding (categorical), StandardScaler (continuous).	✓
10d. Model performance	Methods: Model Evaluation	5–6	AUROC, sensitivity, specificity, precision, F1-score. Calibration: Brier score + calibration curves. Benchmarking: standard logistic regression.	✓
**METHODS — Model Evaluation**				
11. Model performance measures	Methods: Model Evaluation	5–6	AUROC with 95% CI (bootstrap), sensitivity, specificity, precision, F1-score reported at optimal threshold. Calibration (Brier score) reported for all three models.	✓
12. Model updating	Limitations	18	6–12 month revalidation schedule specified; recalibration triggers defined (AUROC <0.85 or revisit rate shift >1%).	✓
**RESULTS — Participants**				
13a. Participant flow	Results: Baseline	6	48,567 total ED visits; 36,230 met inclusion criteria; 798 (2.2%) revisited within 30 days.	✓
13b. Characteristics	Results: Baseline / Table 1	6–7	Baseline characteristics tabulated for revisit vs. non-revisit groups including demographics, vital signs, comorbidities, procedures.	✓
13c. Missing data	Methods: Data Preprocessing	4–5	Missing data rates <5% for all key variables; imputation method stated.	✓
**RESULTS — Model Performance**				
14a. Model performance	Results: Model Performance / Table 2	7–8	AUROC, sensitivity, specificity, precision, F1-score reported for all three models at optimal cut-off (Table 2). ROC curves in Figure 1.	✓
14b. Model calibration	Results: Calibration	11–12	Brier scores: XGBoost 0.169, ElasticNet 0.187, Random Forest 0.037. Calibration	✓

Item	Section	Page	Where Reported / Comment	✓
	Analysis / Figure 8		curves presented as Figure 8.	
**DISCUSSION**				
15a. Interpretation	Discussion	12–18	Results interpreted in context of prior literature; key predictors clinically interpreted via SHAP; incremental ML gain discussed.	✓
15b. Limitations	Policy Implications and Study Limitations	17–18	Single-center, 30-day window, unplanned revisit definition, low precision (PPV 0.09), calibration limitations, temporal generalizability. All explicitly stated.	✓
15c. Generalizability	Discussion / Future Research	17–19	External validation in other medically underserved settings identified as next step. NEDIS benchmark comparison provided.	✓
**OTHER INFORMATION**				
16. Supplementary information	Supplementary	—	Supplementary Figure S1 (logistic regression ROC comparison), Supplementary Figure S2 (SHAP stability heatmap), TRIPOD checklist.	✓
17. Funding	Declarations	—	No external funding received.	✓
18. Registration	—	—	Retrospective cohort study; not applicable for prospective trial registration.	N/A
19. Ethical approval	Ethics Approval	6	IRB approved: Gangneung Asan Hospital IRB No. GNAH 2025-06-001. Informed consent waived (retrospective).	✓
20. Data availability	Declarations	—	Data available from the corresponding author upon reasonable request, subject to institutional data governance.	✓

✓ = Reported   N/A = Not applicable

Note: This checklist follows the TRIPOD (Transparent Reporting of a Multivariable Prediction Model for Individual Prognosis or Diagnosis) statement. Items are mapped to the revised manuscript (Manuscript_Revised_Highlighted.docx). Page numbers refer to the submitted manuscript.
